# Synergistic effects of acyclovir and 3, 19- isopropylideneandrographolide on herpes simplex virus wild types and drug-resistant strains

**DOI:** 10.1186/s12906-015-0591-x

**Published:** 2015-03-11

**Authors:** Thongkoon Priengprom, Tipaya Ekalaksananan, Bunkerd Kongyingyoes, Supawadee Suebsasana, Chantana Aromdee, Chamsai Pientong

**Affiliations:** Department of Microbiology, Faculty of Medicine, Khon Kaen University, Khon Kaen, Thailand; Department of Pharmacology, Faculty of Medicine, Khon Kaen University, Khon Kaen, Thailand; Faculty of Pharmacy, Thammasat University, Bangkok, Thailand; Department of Pharmaceutical Chemistry, Faculty of Pharmaceutical Sciences, Khon Kaen University, Khon Kaen, Thailand; HPV & EBV and carcinogenesis Research Group, Khon Kaen University, Khon Kaen, Thailand

## Abstract

**Background:**

An andrographolide analogue, 3, 19-isopropylideneandrographolide (IPAD), exerts an inhibitory effect on replication of wild-type herpes simplex virus serotype 1 (HSV-1). In this study, we examined the anti-viral activity of IPAD on HSV wild types (HSV-1 strain KOS and HSV-2 clinical isolate) and HSV-1 drug-resistant strains (DRs). Synergistic effects of IPAD with acyclovir (ACV) were also evaluated.

**Methods:**

MTT and cytopathic effect (CPE) reduction assays were performed to determine cytotoxicity and anti-viral activities, respectively. A combination assay was used to determine synergistic effects of IPAD and ACV. Presence of viral DNA and protein in experimental cells was investigated using the polymerase chain reaction and western blotting, respectively.

**Results:**

A non-cytotoxic concentration of IPAD (20.50 μM) completely inhibited CPE formation induced by HSV wild types and HSV-1 DRs after viral entry into the cells. The anti-HSV activities included inhibition of viral DNA and protein synthesis. The minimum inhibitory concentrations of ACV for HSV wild types and HSV-1 DRs were 20.20 and 2,220.00 μM, respectively. Combination of ACV with IPAD showed synergistic effects in inhibition of CPE formation, viral DNA and protein synthesis by HSV wild types as well as HSV-1 DRs. For the synergistic effects on HSV wild types and HSV-1 DRs, the effective concentrations of ACV were reduced.

**Conclusions:**

These results showed the inhibitory potential of IPAD on HSV wild types and HSV-1 DRs and suggested that IPAD could be used in combination with ACV for treatment of HSV-1 DRs infections.

## Background

Herpes simplex viruses (HSV) are common human pathogens that cause herpes labialis, herpes genitalis, keratitis and encephalitis. According to epidemiological surveys, the HSV infection rate has continuously increased in most countries [1]. Nucleoside analogs, acyclovir (ACV) and others such as penciclovir, valaciclovir, and famciclovir, have been approved for treatment of HSV infections worldwide, but severe side effects may occur [2-4]. Moreover, long-term prophylactic and curative ACV treatments may result in the emergence of HSV drug resistance (DRs), especially among immunocompromised individuals such as HIV-infected patients and transplant recipients. Resistance of HSV to ACV has been reported in 5-30% of cases, mainly among immunocompromised patients and particularly in allogeneic bone marrow transplant patients [5]. ACV is an analogue of guanosine that requires activation through triphosphorylation. The first phosphorylation is mainly achieved by HSV thymidine kinase (TK), encoded by the UL23 gene, whereas subsequent phosphorylations are carried out by host cellular kinases [6]. The ACV active form is then incorporated by the viral DNA polymerase, encoded by the UL30 gene, and finally disrupts viral genome replication by a chain-termination mechanism. In accordance with this mechanism of action, viral mutations conferring resistance to ACV have been mapped both in UL23 and UL30 genes, but 95% of HSV strains exhibiting resistance to ACV harbor mutations within the UL23 gene alone. These mutations lead to the production of TK with deficient or altered phosphorylation activity.

 There is an urgent need for cheap, less toxic alternative agents to control and prevent HSV infection and its transmission. Andrographolide (Androg) is a bioactive *ent-*labdane diterpene isolated from *Andrographis paniculata* Nees, Acanthaceae and has been used to treat various diseases traditionally in the Southeast Asian countries, India and China. The pure form of Androg and its derivatives were isolated and already characterized [7]. In our recent studies, 3, 19-isopropylideneandrographolide (IPAD), an analogue of Androg, was found to exert an absolute inhibitory effect on HSV-1 post-infection at the concentration of 11.96 μM [7,8]. IPAD affects the early steps of DNA replication in HSV-1. Its action influences viral DNA synthesis and expression of gC and gD [7].

ACV and IPAD have different structures. This likely means that their modes of action also differ. In this study, IPAD and IPAD in combination with ACV were tested for anti-viral activity against HSV wild types (HSV-1 and HSV-2) and HSV-1 drug-resistant strains (DRs).

## Methods

### Cell line

An African green monkey kidney cell line (Vero) was maintained in Dulbecco’s Modified Eagle Medium (DMEM; Gibco-BRL, Gaithersburg, MD, USA) supplemented with 10% fetal bovine serum (FBS; Gibco-BRL, Gaithersburg, MD, USA), 100 units/ml penicillin G, 100 μg/ml streptomycin, 40 μg/ml gentamicin and 0.25 μg/ml amphotericin B.

### Viruses

HSV wild types used in this study were HSV-1 strain KOS and HSV-2 clinical isolate (kindly provided by Prof. Pilaipan Puthavathana, Mahidol University, Thailand). HSV-1 DRs, kindly provided by Prof. Donald Coen (Biological Chemistry & Molecular Pharmacology, Harvard Medical School, Boston, USA.), were ACGr4 (acyclovir-resistant with thymidine kinase (TK)-deficient), dlsptk (acyclovir-resistant with TK-deletion) and dxpIII (phosphonoacetic acid and phosphonoformate-resistant). The viruses were propagated on Vero cells and viral titers were determined by plaque assays on Vero cells. Aliquots of viral stock were stored at -80°C until use.

### Compound

IPAD was isolated and its structure was identified by a team at the Faculty of Pharmaceutical Sciences, Khon Kaen University, using the procedures described previously [7,8]. The final concentration of DMSO was less than 0.1% and had no toxic effects on the cells.

### Cytotoxicity assay

The effect of IPAD and ACV on cell viability was determined by MTT assay using 3-(4,5-dimethylthiazole-2-yl)-2,5-diphenyltetrazolium bromide (MTT) (SIGMA® (Sigma-Aldrich, Saint Louis, Missouri, USA). Vero cells were seeded in 96-well tissue-culture plates (10^4^ cells/well) and grown at 37°C for 1 day. The culture media were replaced with fresh media containing various concentrations of IPAD (7.80 to 820.00 μM) and ACV (50 to 6,400 μM). After 72 h incubation, the media were replaced with 15 μl of 5 mg/ml MTT in each well and the cells were incubated at 37°C for 4 h. DMSO was added and incubated at room temperature for 15 min. The optical density (OD_540_ nm) was measured by a microplate reader (Tecan-Sunrise-Microplate Reader). Cell viability of the treated cells was calculated by percentage of mean value of the OD_540_ nm compared with the cell control, which was set at 100%. Dose-response curves of three independent experiments were plotted to calculate the 50% cytotoxic concentration (CC50) of IPAD and ACV.

### Anti-HSV activity at the pre-entry step

Vero cells were seeded in 96-well tissue-culture plates (10^4^ cells/well) and grown at 37°C for 1 day. HSV was incubated with IPAD at 37°C for 1 h. Then the mixture was added to Vero cells at 0.01 MOI of HSV and incubated at 37°C for 1 h. After removing the mixture, the cells were washed three times with PBS and incubated in media containing 0.8% carboxymethyl cellulose (CMC). Dextran sulfate (10 μM) was used as a positive control. Cytopathic effect (CPE) was observed under an inverted microscope at 48 and 72 h post-infection (h.p.i.). The foci of CPE were counted under an inverted microscope and compared with the virus control at 72 h.p.i.

### Anti-HSV activity at the post-entry step

Vero cells were seeded in 96-well tissue-culture plates (10^4^ cells/well) and grown at 37°C for 1 day. HSV at 0.01 MOI were adsorbed on Vero cells at 37°C for 1 h. After the unadsorbed viruses had been removed, the infected cells were incubated in media containing 0.8% CMC and IPAD at 37°C for 72 h. ACV (20.20 μM) was used as the positive control. The foci of CPE were counted under an inverted microscope and compared with the virus control at 72 h.p.i. The cells were harvested for DNA and protein extraction.

### HSV detection by polymerase chain reaction (PCR)

Total DNA was extracted from the tested cells using the PUREGENE DNA purification system (PUREGENE®; Gentra Systems Inc., Minnesota, USA). HSV DNA polymerase gene, UL30 was amplified using a forward primer (GTGTTGTGCCGCGGTCTCAC) and a reverse primer (GGTGAACGTCTTTTCGAACTC) with an expected product size about 123 bp. A housekeeping gene, β-actin was used as the internal control and was amplified using a forward primer (TCACCCACACTGTGCCCATCTACG) and a reverse primer (CAGCGGAACCGCTCATTGCCAATG) with an expected product size about 294 bp. The amplified PCR product was visualized by electrophoresis in a 1.5% agarose gel.

### HSV detection by western blotting

The tested cells were lysed with lysis buffer and proteins were extracted. The presence of HSV-1 glycoprotein D (gD) in the extracted protein was determined by western blotting using a primary polyclonal antibody against gD (kindly provided by Prof. Gary H. Cohen and Prof. Roselyn J. Eisenberg, University of Pennsylvania, Philadelphia, Pennsylvania, USA and the secondary antibody, horseradish peroxidase (HRP)-conjugated goat anti-rabbit immunoglobulin G (Zymed®; Invitrogen Co., California, USA) according to the protocol of the previous study [8].

### Evaluation of drug synergy

Potential synergistic effects of ACV and IPAD on HSV infections were evaluated at the post-entry step using the CPE reduction assay. Each drug alone, or their combination, was tested at the equipotency ratio based on its corresponding IC_100_, IC_50_ and IC_10_ values. The synergistic effect of ACV and IPAD was calculated by using a combination index (CI) as described previously [9]. A CI value of 1 indicates an additive effect; CI < 1 indicates a synergistic effect, and CI > 1 indicates an antagonistic effect. Viral DNA and protein were determined using PCR and western blotting to confirm the inhibitory effect.

## Results

### Cytotoxicity and anti-HSV activity of IPAD

The maximum concentration of IPAD which did not affect Vero cell viability was 22.55 μM while the highest dose of ACV used in the experiment (6,400 μM) did not affect Vero cell viability. The CC_50_ of IPAD and ACV were 39.71 μM and >6,400 μM, respectively (Table 1).

**Table 1:** **Cytotoxic effect, anti-viral activity and selective index of IPAD and ACV at the post-entry step**

	**CC**_**50**_^**a**^ **(μM)**	**Anti-viral activity** ^**b**^ **against**									
		**HSV-2**		**HSV-1 KOS**		**HSV-1 ACGr4**		**HSV-1 dlsptk**		**HSV-1 dxpIII**	
		**IC**_**50**_ **(μM)**	**SI** ^**c**^	**IC**_**50**_ **(μM)**	**SI** ^**c**^	**IC**_**50**_ **(μM)**	**SI** ^**c**^	**IC**_**50**_ **(μM)**	**SI** ^**c**^	**IC**_**50**_ **(μM)**	**SI** ^**c**^
**IPAD**	39.71	18.01	2.20	16.96	2.34	17.89	2.22	16.86	2.36	17.12	2.32
**ACV**	>6,400	0.80	>8,000	0.49	>13,061	575.91	>11.11	450.25	>14.21	161.45	>39.64

The value represents mean of three independent experiments.

^a^Cytotoxic effect was determined using the MTT assay. CC_50_ was the concentration reducing cell viability by 50% relative to controls.

^b^Anti-viral activity was determined using the CPE reduction assay. IC_50_ was the concentration that reduced HSV-induced CPE formation by 50% relative to controls.

^c^SI (selective index) was calculated as the ratio of CC_50_ to IC_50_.

**Figure 1:**
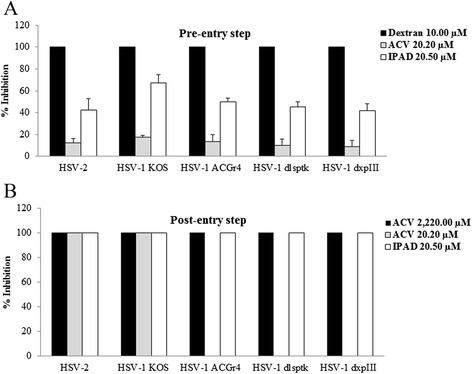
**Effects of IPAD and ACV on HSV wild types and HSV-1 DRs determined using the CPE reduction assay. A**: pre-entry step. **B**: post-entry step.

To determine the anti-viral activity of IPAD against HSV wild types and HSV-1 DRs at the pre- and post-entry steps, a CPE reduction assay was performed. At the pre-entry step, 10 μM dextran sulfate (positive control) exhibited 100% inhibition whereas 20.50-22.55 μM IPAD exerted about 50-60% inhibition against both HSV wild types and HSV-1 DRs. In contrast, ACV had little anti-HSV activity at the pre-entry step (Figure 1A). In the present study, we also performed virus binding and internalization assays separately and the results showed that there were no significant effects in these steps of the virus multiplication cycle (data not shown). At the post-entry step (Figure 1B), the lowest concentrations of IPAD and ACV which completely inhibited CPF formation by HSV wild types were 20.50 μM and 22.20 μM, respectively. IPAD at 20.50 μM also exerted 100% inhibition against HSV-1 DRs while for ACV, a concentration of at least 2,220.00 μM was required to exert complete inhibition against HSV-1 DRs.

IC_50_ values of IPAD, which were determined at the post-entry step using the CPE reduction assay, were 17-18 μM according to the target virus strains as shown in Table 1. Interestingly, the IC_50_ as well as SI values of IPAD against HSV wild types and HSV-1 DRs were not different (Table 1). In contrast, the IC_50_ and SI values of ACV against HSV wild types and HSV-1 DRs were markedly different. The IC_50_ values for ACV against HSV wild types were less than 1.0 μM, whereas those against HSV-1 DRs were more than 160 μM (Table 1).

**Figure 2:**
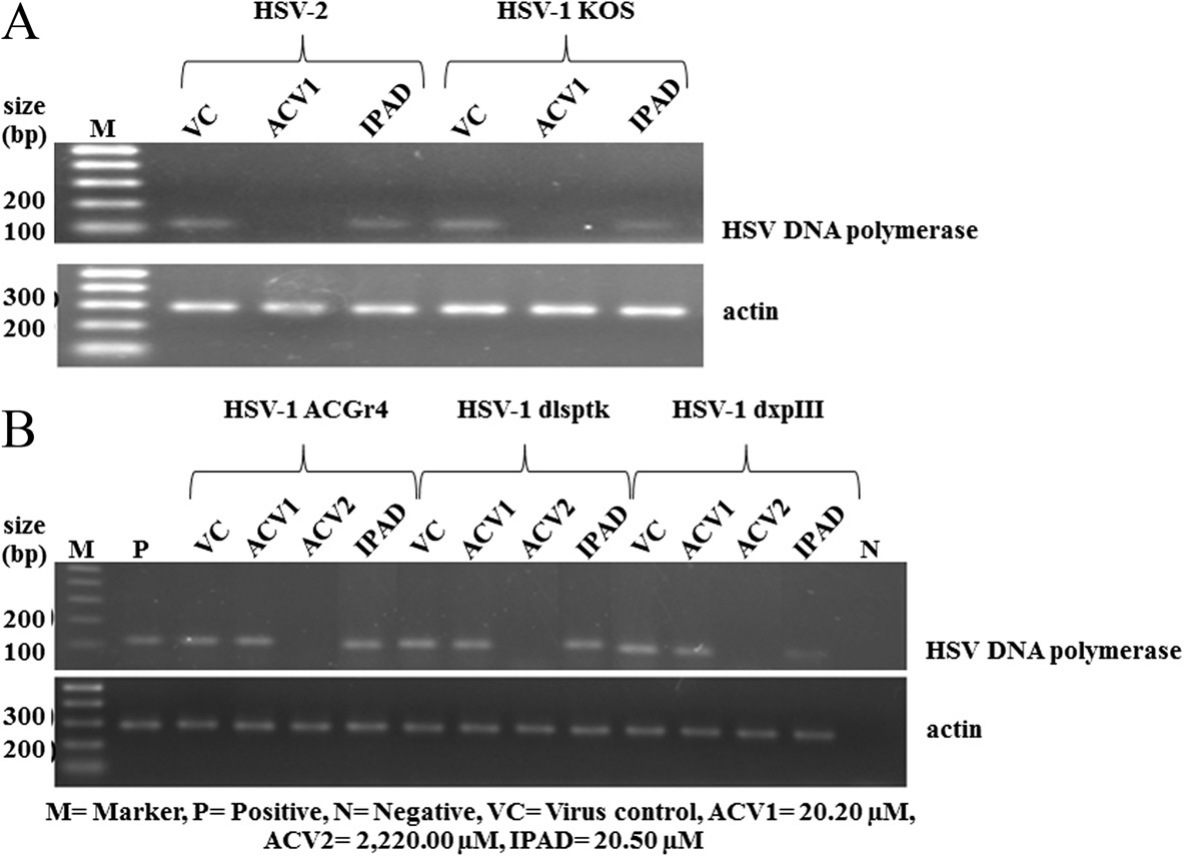
**Effects of IPAD and ACV on HSV DNA synthesis in the post-infection treatment with IPAD and ACV for 72 h. A**: HSV wild types including HSV-1 KOS and HSV-2 clinical isolate. **B**: HSV-1 DRs including HSV-1 ACGr4, HSV-1 dlsptk and HSV-1 dxpIII. M = marker, P = positive control, VC = virus control, N = negative control.

**Figure 3:**
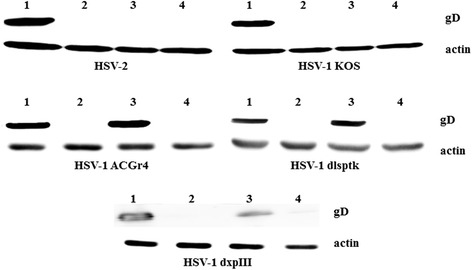
**Effects of IPAD and ACV on HSV protein expression in the post-infection treatment with IPAD and ACV for 72 h.** Treated and untreated cells were analyzed by SDS–PAGE/western blotting using specific antibodies for viral (gD) and cellular (β-actin) proteins. Lane 1: viral control; Lane 2: HSV-infected cells treated with IPAD (20.50 μM); Lane 3: HSV-infected cells treated with ACV (20.20 μM); Lane 4: cellular control.

To confirm the inhibitory effect of IPAD against both HSV wild types and HSV-1 DRs at the post-entry step, the presence of HSV DNA and protein in infected cells was examined using PCR and western blotting. Even though IPAD exhibited 100% inhibition of CPE formation, a small amount of viral DNA was detected at 72 h.p.i. (Figure 2). In contrast, ACV showed complete inhibition of viral DNA synthesis. However, IPAD and ACV significantly suppressed the expression of viral protein as shown in Figure 3. This result suggests that the inhibitory effect of IPAD may be exerted at an early step of gene expression and involve a target different from that of ACV.

### Synergistic effects of acyclovir and IPAD on HSV infections

The combined effect of ACV and IPAD on HSV infections was examined at the post-entry step using the CPE reduction assay and confirmed by PCR and western blotting. Table 2 shows synergistic effects and CI values of ACV and IPAD combined at different concentrations and determined using the CPE reduction assay. When IC_10_ concentrations of ACV and IPAD were used in the combination assay, they still exerted complete inhibition against HSV wild types and HSV-1 dxpIII infections. CI values < 1 indicate synergism between these two compounds. When used in combination with IPAD, the concentration of ACV required for complete inhibition of HSV-1 dxpIII was reduced from 2,220.00 to 18.70 μM. Moreover, in the combination assay, IC_50_ values for ACV and IPAD were reduced more than 10 times when compared with a single drug assay (Table 2). This result indicated the increased SI of IPAD from 2 (the single drug assay) to more than 20 (the combination assay).

**Table 2:** **Synergistic effects of ACV and IPAD on HSV infection**

**Compounds combination ratio**	**Compound concentration (μM)**		**% Inhibition of IPAD + ACV**	**Experimental CI**	**Description**
	**IPAD**	**ACV**			
**HSV-2**					
	20.50	-	100		
	-	20.20	100		
**IC**_**100**_ **xIC**_**100**_	20.50	20.20	100		
**IC**_**50**_ **xIC**_**50**_	18.01	0.80	100		
**IC**_**10**_ **xIC**_**10**_	15.00	0.04	100	<1	synergism
	18.01	0.0008	50		
	0.004	0.80	50		
**HSV-1 KOS**					
	20.50	-	100		
	-	20.20	100		
**IC**_**100**_ **xIC**_**100**_	20.50	20.20	100		
**IC**_**50**_ **xIC**_**50**_	16.96	0.49	100		
**IC**_**10**_ **xIC**_**10**_	13.87	0.02	100	<1	synergism
	16.96	0.008	50		
	0.009	0.49	50		
**HSV-1 ACGr4**					
	20.50	-	100		
	-	2,220.00	100		
**IC**_**100**_ **xIC**_**100**_	20.50	2,220.00	100		
**IC**_**50**_ **xIC**_**50**_	17.89	575.91	100		
**IC**_**10**_ **xIC**_**10**_	14.96	25.36	88.5	<1	synergism
	17.89	58.16	50		
	0.89	575.91	50		
**HSV-1 dlsptk**					
	20.50	-	100		
	-	2,220.00	100		
**IC**_**100**_ **xIC**_**100**_	20.50	2,220.00	100		
**IC**_**50**_ **xIC**_**50**_	16.86	450.25	98.55		
**IC**_**10**_ **xIC**_**10**_	15.00	23.12	88.41	< 1	synergism
	16.86	4.51	50		
	0.85	450.25	50		
**HSV-1 dxpIII**					
	20.50	-	100		
	-	2,220.00	100		
**IC**_**100**_ **xIC**_**100**_	20.50	2,220.00	100		
**IC**_**50**_ **xIC**_**50**_	17.12	161.45	100		
**IC**_**10**_ **xIC**_**10**_	14.90	18.70	100	< 1	synergism
	17.12	0.16	50		
	0.009	161.45	50		

**Figure 4:**
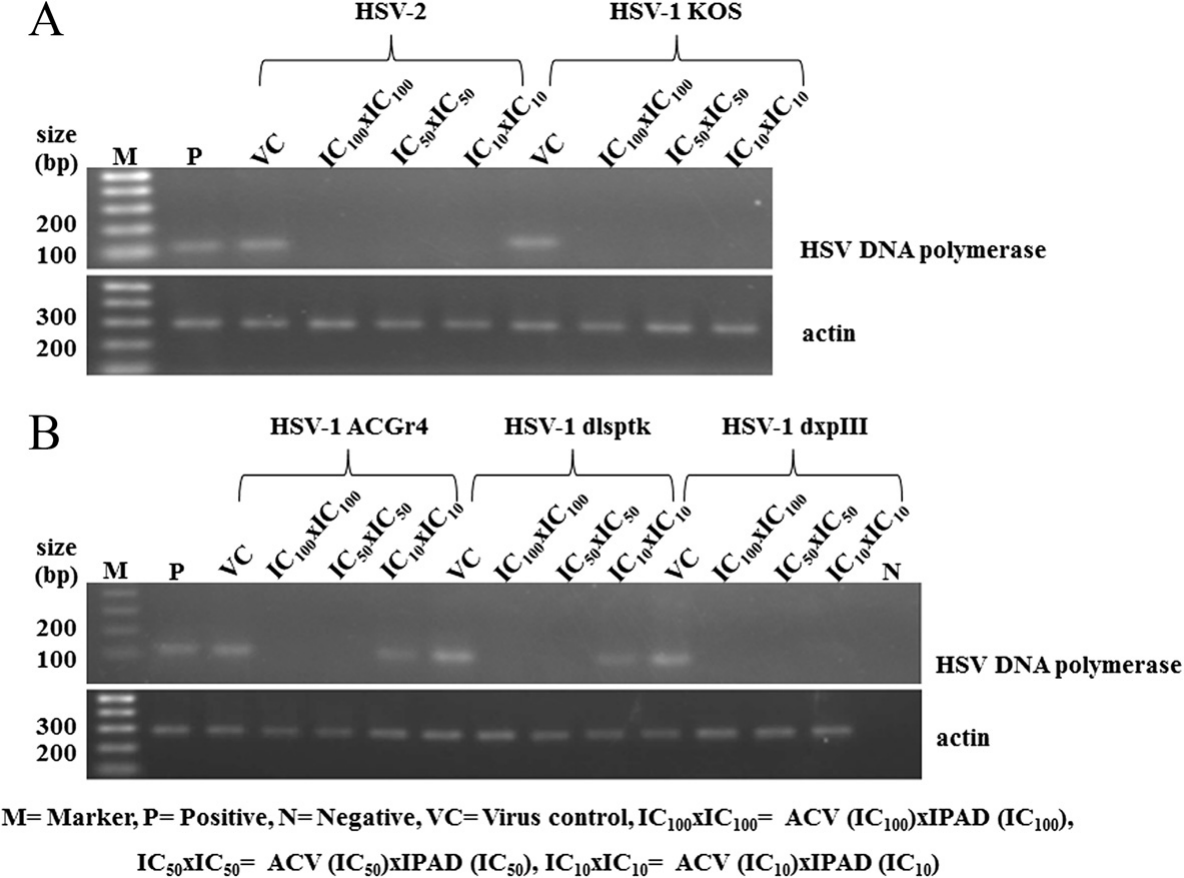
**Combined effects of IPAD and acyclovir on HSV DNA synthesis.** Vero cells infected with HSV wild types and HSV-1 DRs were treated with IPAD and ACV in various concentrations for 72 h and analyzed by PCR using specific primers for viral (HSV DNA polymerase) and cellular (β-actin) genes. **A**: HSV wild types. **B**: HSV-1 DRs.

**Figure 5:**
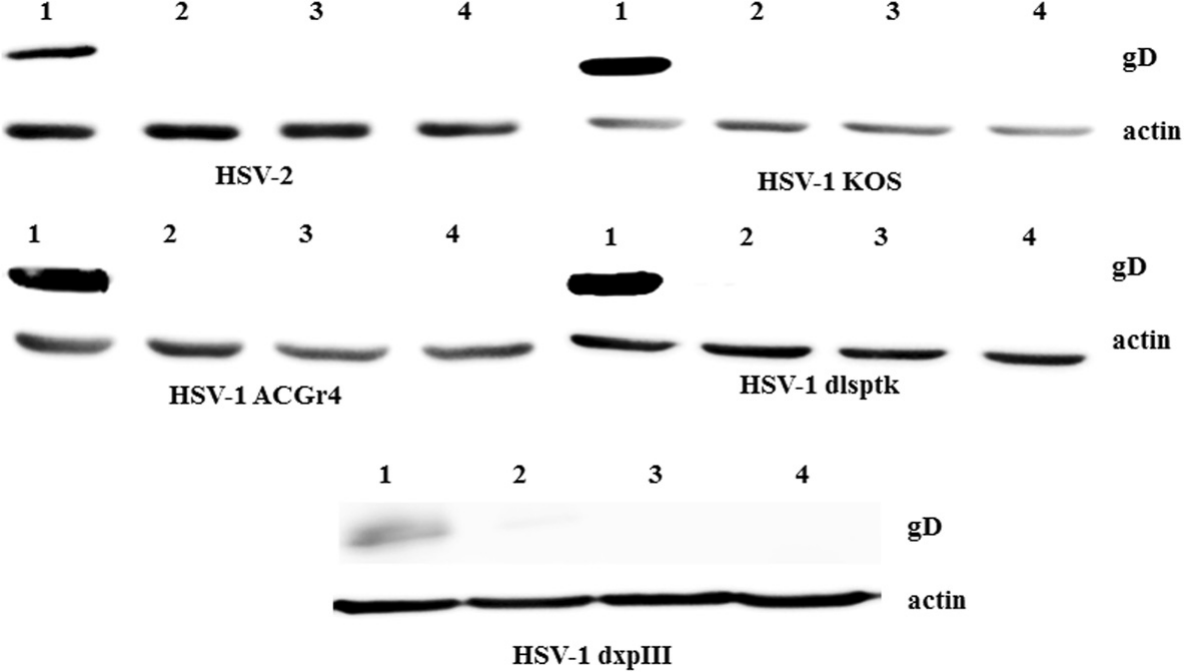
**Combined effects of IPAD and acyclovir on HSV protein synthesis.** Vero cells infected with HSV wild types and HSV-1 DRs were treated with IPAD combined with ACV in various concentrations for 72 h and analyzed by SDS–PAGE/western blotting using specific antibodies for viral (gD) and cellular (β-actin) proteins. Lane 1: untreated HSV-infected Vero cell control; Lane 2: HSV-infected cells treated with ACV (IC_100_) and IPAD (IC_100_); Lane 3: HSV-infected cells treated with ACV (IC_50_) and IPAD (IC_50_); Lane 4: HSV-infected cells treated with ACV (IC_10_) and IPAD (IC_10_).

The synergistic effects of ACV and IPAD against HSV wild types and HSV-1 DRs were confirmed by testing for the presence of viral DNA and protein in experimental cells. Viral DNA synthesis was completely inhibited when IPAD and ACV were combined, each at their IC_50_ concentration (Figure 4). These concentrations also completely inhibited production of the viral protein according to western blotting (Figure 5).

## Discussion

This study demonstrated the inhibitory effects of IPAD, a semi-synthetic compound derived from Androg, on HSV wild types and HSV-1 DRs. The effects included inhibition of CPE formation, viral protein, gD synthesis and viral DNA synthesis. IPAD was spectacularly effective in inhibiting HSV-1 DRs, presumably involving a mode of action different from ACV or phosphonoacetate. The combination of ACV with IPAD produced synergistic effects, reducing concentrations of both compounds required for complete inhibition of DNA replication and late protein synthesis of HSV-1 wild types and HSV-1 DRs at the post-entry step of infection.

The anti-viral effects of IPAD against HSV-1 KOS (wild type) at the virus replication and releasing stages was previously reported [8]. In the present study, anti-viral activity of IPAD against HSV wild types and HSV-1 DRs was evaluated. IPAD showed higher cytotoxicity towards Vero cells than did ACV. However, IPAD at 20.50 μM, which was not cytotoxic to Vero cells, displayed anti‐viral effect at the post-entry step against HSV wild types and HSV-1 DRs (Figure 1B) corresponding with the previous report [8]. This was confirmed by demonstration of significant reduction of HSV DNA and protein synthesis in all HSV strains (Figures 2 and 3). Interestingly, IPAD exhibited highly anti-herpetic activity against HSV-1 DRs, not only the acyclovir-resistant strains (HSV-1 ACGr4 and HSV-1 dlsptk), but also the phosphonoacetic acid-resistant strain (HSV-1 dxpIII). This result suggests that the inhibitory targets of IPAD are probably different from those of acyclovir and phosphonoacetic acid. Recently, some compounds that showed an inhibitory effect on HSV drug-resistant strains were reported. Glucoevatromonoside, a cardenolide isolated from a Brazilian cultivar of *Digitalis lanata*, was reported to inhibit HSV-1 KOS, HSV-1 29R (acyclovir-resistant) and HSV-2 333 by the inhibition of viral proteins synthesis [10]. Oxyresveratrol, derived from a Thai medicinal plant, inhibited HSV replication and viral late protein synthesis with pretreatment in a one-step growth assay of HSV-1 and HSV-2 [11]. The inhibitory effects of plant-derived polyphenolic compounds, castalagin, vescalagin and grandinin (C-glucosidic ellagitannins containing nonahydroxyterphenoyl), on the replication of ACV-resistant strains of HSV-1and HSV-2 were shown in MDBK cells [12]. The anti-viral activity of Androg or its derivatives against other viruses has also been reported. Androg effectively inhibited the expression of Epstein–Barr virus (EBV) lytic proteins, Rta, Zta and EA-D, during the viral lytic cycle in P3HR1 cells. Transient transfection analysis revealed that the lack of expression of Rta, Zta and EA-D is caused by the inhibition of the transcription of BRLF1 and BZLF1, two EBV immediate early genes that encode Rta and Zta, respectively [13]. The 14-α-lipoyl andrographolide (AL-1) could directly interfere with an influenza hemagglutinin to block binding to cellular receptors in a potentially new and potent mechanism of action [14].

Some studies have reported that the combination of oxyresveratrol and ACV exhibited a synergistic effect against HSV-1 [11] and that the combination of ellagitannin(s) and ACV exhibited a much stronger synergistic effect against ACV-resistant HSV-1 compared to ACV-resistant HSV-2 [12]. In the present study, the potential synergistic effects between IPAD and acyclovir were also observed against both HSV wild types and HSV-1 DRs (Table 2). In addition, this synergistic effect not only reduced the effective concentration of ACV but also of IPAD and increased the SI value for IPAD from 2 to 20.

ACV is a nucleoside analog that exhibits anti-herpetic activity after phosphorylation by viral TK. The acyclovir triphosphate then interferes with viral DNA polymerization through competitive inhibition with guanosine triphosphate and obligatory DNA chain termination [3]. Base on the inhibitory effect of IPAD on viral DNA and protein synthesis of HSV-1 DRs (Figures 4 and 5), this study suggested that IPAD and ACV have different targets. Therefore, the combined application of IPAD and ACV should delay the development of drug (ACV) resistance. Lower doses of ACV and IPAD, possible when these are used in combination, can also reduce any cytotoxic effects of the drugs.

## Conclusions

In summary, the anti-viral activity of IPAD likely involves a mode of action different from ACV or phosphonoacetate. The combination of IPAD with ACV could reduce the concentration of ACV required for treatment and suggests that IPAD can be used in combination with ACV for inhibition of herpes infections, especially HSV-1 DRs.
